# Cancer morbidity and mortality trends in Trinidad and Tobago (2008–2018)

**DOI:** 10.1186/s41043-023-00395-1

**Published:** 2023-06-27

**Authors:** Chavin D. Gopaul, Aruna Singh, Akil Williams, Dale Ventour, Davlin Thomas

**Affiliations:** 1 North Central Regional Health Authority, Ground Floor, Building 7, Eric Williams Medical Science Complex, Uriah Butler Highway, West Indies, Port of Spain, Trinidad and Tobago; 2 North West Regional Health Authority, Dundonald Street, West Indies, Port of Spain, Trinidad and Tobago; 3 Caribbean Centre for Health Systems Research and Development, University of the West Indies, St. Augustine, Trinidad and Tobago; 4 University of the West Indies, St. Augustine Campus, West Indies, St. Augustine, Trinidad and Tobago; 5 North Central Regional Health Authority, Third Floor, Building 39, Eric Williams Medical Science Complex, Uriah Butler Highway, West Indies, Port of Spain, Trinidad and Tobago

**Keywords:** Cancer trends, Cancer morbidity, Cancer mortality, Cancer incidence, Cancer registry, Trinidad and Tobago

## Abstract

**Purpose:**

Cancer is a leading cause of death in the Caribbean, and the Republic of Trinidad and Tobago is no exception. Evidence suggests that cancer incidence and mortality may vary based on demographic factors across the different cancer types. This study aimed to investigate the incidence and mortality trends associated with cancer cases in Trinidad and Tobago for the period 2008–2018, across different age groups, gender, and ethnicity.

**Methods:**

Data on 15,029 incident cancer cases were reported to the Dr. Elizabeth Quamina Cancer Registry between 2008 and 2018. The retrospective data were analyzed by sex, ancestry, and age, and were reported using Trinidad and Tobago population statistics for the period 2008–2018.

**Results:**

The incidence of prostate and breast cancers was high among males and females, respectively. Among males, the highest cancer mortality was associated with prostate, lung, colon, blood, and pancreatic cancers, respectively. Among females, the highest cancer mortality was associated with breast, ovary, colon, blood, and pancreatic cancers. The frequency of occurrence of the top five cancer sites was the highest among Afro-Trinidadians followed by Indo-Trinidadians. Most females diagnosed with breast cancer were at a localized stage, while most males diagnosed with breast cancer were at a distant or regional stage. Most individuals diagnosed with blood cancer were at a distant stage. For lung and colon cancer, the stage of diagnosis for most males and females was either distant or unknown. Majority of males are diagnosed with prostate cancer at an unknown stage.

**Conclusions:**

The findings indicate highest cancer incidence and mortality occur among Afro-Trinidadians. The stage at diagnosis varies across cancer types and gender.

**Supplementary Information:**

The online version contains supplementary material available at 10.1186/s41043-023-00395-1.

## Introduction

Cancer is considered the leading cause of death and is responsible for decreasing life expectancy globally [1, 2]. A report by the World Health Organisation (WHO) ranks cancer as the first or second cause of death among individuals aged 70 years or less among 112 countries [3]. The GLOBOCAN 2018 database, data compiled by the International Agency for Research on Cancer, suggested that there were about 18.1 million new cancer cases globally in 2018 [1]. The analysis also showed that the number of deaths from cancer in 2018 was 9.6 million [1]. Sung et al. [2], in their analysis of the GLOBOCAN database, noted that in the year 2020, there were an estimated 19.3 million new cases of cancer and 10 million deaths. The increasing burden of cancer incidence and mortality is associated with social-economic development [2, 4]. Torre et al. argued that cancer incidence is expanding across countries of various income levels due to the growth and aging of the population [5] (Additional file 1).

Demographic factors such as gender have been suggested to influence the incidence and mortality associated with cancer [2]. According to Ferlay et al. [1], the type of cancer with highest incidence among males and females is lung cancer. Among males, prostate cancer is the most common [1, 2]. Females have a higher incidence of breast cancer [1, 2]. In men, lung cancer is associated with high mortality rates followed by liver cancer and stomach cancer [1]. Among females, the highest mortality is associated with breast cancer, followed by lung cancer, cervical cancer, and stomach cancer, respectively [1].

Cancer incidence and mortality rate are also associated with age. Chen et al. noted that the cancer incidence rate is lower among individuals aged 39 years and below [6]. The incidence of cancer is highest among individuals aged between 80 and 85 years [6]. According to Wang et al. [7], the incidence of liver cancer is 23.44 times and 27.28 times higher in males and females, respectively, aged 90–94 years compared to those aged 20–24 years. Concerning prostate cancer, Siegel et al. [8] noted that the highest incidence occurs among men aged 70–74 years.

Cancer is a leading cause of death in the Caribbean, and the Republic of Trinidad and Tobago is no exception [9]. The ancestral composition of the estimated 1.4 million population is diverse, with Trinidad’s population comprised of 37.01% East Indian ancestry, 31.76% African ancestry, 23.52% Mixed ancestry, and < 1% Chinese, White, and Syrian/Lebanese ancestry, and Tobago’s population consisting of mostly African ancestry (85.29%) [10, 11]. The customs and traditions that the diverse ethnic composition brings with it are reflected in the sociocultural landscape of the country [12]. Evidence suggests that cancer incidence and mortality may vary based on demographic factors across the different cancer types. Research on the epidemiology of cancer in Trinidad and Tobago as it relates to environmental, lifestyle, and demographic factors, including ancestry, is thus important in informing health programs and policies surrounding cancer. Understanding the incidence and mortality associated with cancer is important in the development of targeted interventions [5]. Previous research on cancer incidence and mortality in the country has been reported for the period 1995–2009 based on population data from the Dr. Elizabeth Quamina Cancer Registry, established as the National Cancer Registry of Trinidad and Tobago in 1994 [13]. There is therefore a need to update the existing literature concerning the cancer mortality and morbidity trends in the country. In this study, the objective was to estimate the cancer incidence and mortality across different age groups, gender, and ethnicity, from 2008 to 2018, in Trinidad and Tobago.

## Methodology

The authors collected retrospective data from the National Cancer Registry of Trinidad and Tobago (NCRT&T) which is the national repository for all cancer data produced by the country. The dataset obtained from the NCRT&T contained age, sex, ethnicity, stage, grade, method of cancer detection and treatment, cancer site, date of incidence, age at diagnosis and cause of death. Cancer data produced by both public and private healthcare institutions are collected by the registry. Cancer histology is usually coded using WHO International Classification of Diseases of Oncology (ICD-O) code C61.9. Population estimates for the study were utilized from the Trinidad and Tobago Central Statistical Office (CSO) 2000 and 2018. CSO collects several population statistics that include but are not limited to ethnicity, sex and population rates disaggregated by year. The age standardized incidence and mortality rates (per 100,000 TT population) by age, sex, and individual years were calculated. Ethical approval was obtained from the North Central Regional Health Authority (NCRHA) Ethics Committee.

## Data analysis

IBM SPSS Statistics and Microsoft Excel were used for data analysis. Cross-tabulations were used to determine the number of cancer cases by year for the following variables: cancer sites, staging, gender, and ethnicity. The incidence and mortality rates were determined for the period 2008–2018. The incidence rate was determined by dividing the number of cancer cases each year by the mid-year population size for the corresponding year and multiplying the outcome by 100,000. The mortality rate was determined by dividing the number of deaths each year by the mid-year population size for the corresponding year and multiplying the outcome by 100,000.

## Results

### Incidence and mortality among males and females

The top three cancer sites with the highest incidence among men were prostate, lung, and colon for the period 2008–2018. In 2018, the incidence of prostate, lung, and colon cancers was 70.97, 23.90, and 13.49 per 100,000 males, respectively (Table 1).

**Table 1:** Incidence by cancer site per 100,000 by years among men

Years	Cancer site								
	Anal	Blood	Breast	Lung	Colon	Pancreas	Prostate	Rectum	Stomach
2008	1.07	7.77	0.61	17.83	12.65	6.70	59.89	5.33	6.10
2009	0.46	7.31	1.22	20.40	11.72	7.91	57.68	4.72	5.02
2010	0.45	6.81	0.45	15.74	11.20	4.99	60.53	4.09	5.45
2011	0.75	6.45	0.60	16.81	11.11	4.80	51.63	4.20	4.50
2012	0.15	8.36	0.45	14.93	9.40	5.82	52.40	3.58	4.93
2013	0.30	7.29	1.19	17.40	7.58	5.50	45.35	2.08	3.72
2014	0.59	9.19	0.59	18.96	9.04	4.74	46.52	4.44	2.67
2015	0.89	11.22	0.89	20.53	11.67	7.24	56.12	3.54	3.69
2016	0.44	8.83	0.88	15.75	11.34	5.30	50.20	4.56	3.97
2017	0.44	9.84	1.03	21.89	9.26	6.91	59.65	3.67	3.23
2018	0.44	6.89	1.32	23.90	13.49	7.04	70.97	7.04	4.40

The top three cancer sites with the highest mortality during the period 2008–2018 were prostate, lung, and colon. The mortality for prostate, lung, and colon cancers for 2018 was 22.00, 11.58, and 5.28 per 100,000 males, respectively (Table 2). Similar evidence was found for the period 1995–2009, as the highest incidence and mortality rates were seen for prostate and lung cancers among men [5].

**Table 2:** Mortality by cancer site men scale by rate per 100,000 by years

Years	Cancer site								
	Anal	Blood	Breast	Lung	Colon	Pancreas	Prostate	Rectum	Stomach
2008	0.15	4.57	0.00	13.10	5.18	5.18	26.21	2.29	4.57
2009	0.30	3.04	0.30	11.42	4.41	5.33	16.29	1.52	2.28
2010	0.15	2.12	0.15	9.53	4.99	2.57	16.95	1.21	2.72
2011	0.30	2.70	0.15	9.76	3.90	3.00	15.01	1.35	2.70
2012	0.15	3.58	0.15	8.06	2.99	3.58	18.66	1.04	1.79
2013	0.15	2.53	0.45	6.84	2.38	2.68	12.93	1.19	1.78
2014	0.30	2.67	0.00	10.22	2.96	2.52	12.00	1.04	0.89
2015	0.59	4.73	0.15	10.63	4.58	3.54	14.92	1.62	2.07
2016	0.29	2.94	0.29	6.77	4.71	2.50	12.51	1.18	1.91
2017	0.00	4.41	0.29	11.17	3.97	4.70	16.90	1.47	1.03
2018	0.15	2.64	0.29	11.58	5.28	3.96	22.00	2.20	2.64

Breast, colon, and ovarian were the top three cancer sites among women with the highest incidence during the period 2008–2018. The incidence of breast, colon, and ovarian cancer in 2018 was 64.23, 12.55, and 11.96 per 100,000 females, respectively (Table 3).

**Table 3:** Incidence by cancer site among women per 100,000 by years

Years	Cancer site								
	Anal	Blood	Breast	Lung	Colon	Ovary	Pancreas	Rectum	Stomach
2008	0.46	5.37	59.02	5.37	11.34	10.27	5.21	4.14	3.83
2009	0.61	5.82	58.34	6.58	9.80	8.88	4.90	3.52	4.29
2010	0.30	5.63	50.24	5.78	9.44	10.05	4.87	3.35	1.67
2011	0.60	7.25	55.61	7.25	11.64	10.43	4.99	3.17	3.93
2012	0.75	6.76	37.43	5.11	8.42	7.97	3.61	2.25	3.46
2013	0.60	6.14	34.43	4.79	8.98	7.04	4.34	1.80	3.14
2014	0.15	5.67	41.17	6.56	8.65	8.95	4.62	1.94	3.28
2015	0.45	8.18	50.26	7.29	11.60	9.37	5.65	3.42	3.27
2016	0.74	7.71	46.40	7.26	10.08	10.67	4.74	3.11	3.26
2017	0.44	6.07	53.85	6.81	9.17	12.28	5.62	2.37	2.51
2018	1.03	7.83	64.23	7.24	12.55	11.96	5.17	2.21	2.95

The cancer sites associated with the highest mortality among females for the period 2008–2018 were breast, ovarian, and colon. Similarly, during the period 1995–2009, the highest incidence and mortality were observed for breast, cervical, and uterine cancer among women [5]. In 2018, the mortality for breast, ovarian, and colon cancers was 10.93, 5.02, and 4.58 per 100,000 females, respectively (Table 4).

**Table 4:** Mortality by cancer site women scale per 100,000 by years

Years	Cancer site								
	Anal	Blood	Breast	Lung	Colon	Ovary	Pancreas	Rectum	Stomach
2008	0.15	2.91	16.56	4.75	4.29	4.91	3.68	1.38	3.22
2009	0.31	2.14	14.70	3.52	4.44	3.06	2.30	1.07	2.14
2010	0.15	1.98	7.76	3.35	3.20	3.50	2.28	1.07	0.76
2011	0.15	2.27	11.94	3.02	3.78	3.78	2.57	0.91	1.96
2012	0.30	2.86	10.97	2.56	2.25	3.01	1.80	0.30	1.05
2013	0.00	2.10	9.28	1.80	4.34	1.95	2.69	0.75	1.35
2014	0.15	2.09	8.20	2.54	2.54	3.43	2.54	0.75	1.94
2015	0.00	3.12	10.26	3.72	5.06	4.16	3.12	0.89	1.49
2016	0.44	3.11	8.60	3.41	3.26	2.82	1.78	0.89	1.48
2017	0.00	2.22	13.17	3.40	3.11	4.73	3.70	0.44	1.33
2018	0.15	2.81	10.93	3.25	4.58	5.02	3.54	0.44	1.18

### Frequency distribution of cancer by ethnicity

**Fig. 1:**
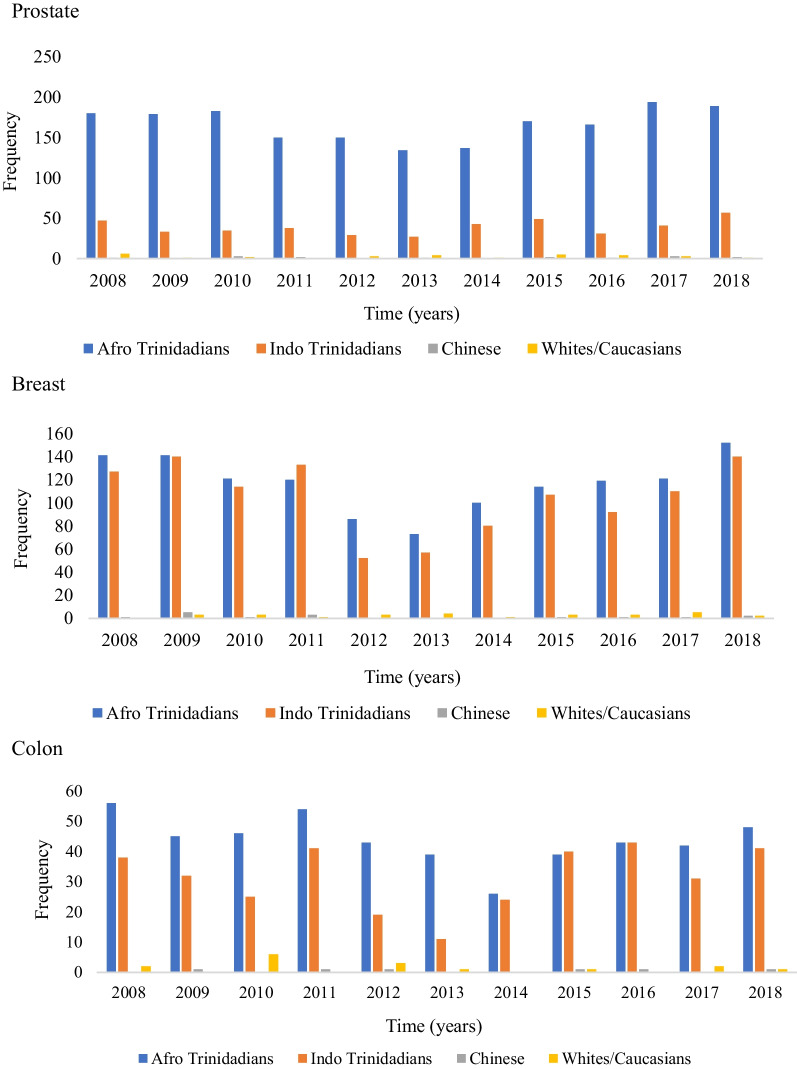

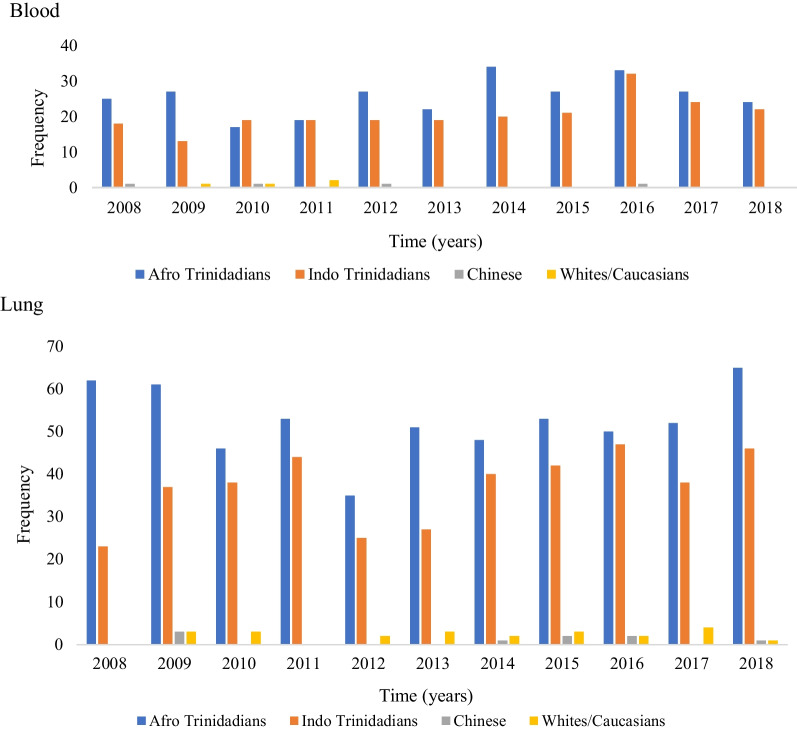
Occurrence of top five cancer sites across the different ethnicities

The frequency distribution of the top five cancer sites (prostate, breast, colon, blood, and lung) by ethnicity were determined for the period 2008–2018. Concerning prostate cancer, Fig. 1 shows that the frequency of occurrence was the highest among Afro-Trinidadians between 2008 and 2018. Indo-Trinidadians had the second-highest frequency of prostate cancer. A similar trend was also observed for the remaining most frequently occurring cancer sites, with Afro-Trinidadians having the highest frequency followed by Indo-Trinidadians.

### Percentage distribution of stage of cancer by sex

As shown in Table 5, most females (79.8%) and males (78.7%) with blood cancer had a distant stage distribution. The findings indicate that most persons (M: 41.7% & F: 43.7%) were diagnosed with a distant stage of lung cancer. For breast cancer, 30.3% of females were at a localized stage, 29.6% were at a regional stage, and 19.1% were at a distant stage. Among males with breast cancer, 30.2% were at a regional stage, 20.6% were at a distant stage, and 17.5% were at a localized stage. Most participants had an unknown stage of colon (M: 37.7% & F: 34.0%) and prostate (47.6%) cancer when diagnosed. Cancers are often diagnosed via metaphases, and during cancer registration, the site of the metaphases is not recorded. Hence, it is documented as an unknown site. It should be noted that cancer metastasized via the staging was recorded as distant.

**Table 5:** Cancer staging at the time of diagnosis between males and females

Sex	Stage	Blood, spleen (%)	Breast (%)	Bronchus, lung (%)	Colon (%)	Prostate (%)
Female	Distant	79.8	19.1	43.7	32.4	
	In situ	0.0	1.5	0.0	0.1	
	Localized	0.0	30.3	7.7	14.9	
	Regional	0.0	29.6	5.8	18.6	
	Unknown	20.2	19.5	42.8	34.0	
Male	Distant	78.7	20.6	41.7	28.4	22.1
	In situ	0.0	1.6	0.0	0.0	0.2
	Localized	0.3	17.5	5.1	15.8	22.7
	Regional	1.5	30.2	6.3	18.0	7.4
	Unknown	19.5	30.2	46.9	37.7	47.6

## Discussion

### Incidence

Demographic factors such as gender have been suggested to influence the incidence of cancer [1, 2]. The findings of this study indicate that the incidence of the major types of cancer by sex increased between 2008 and 2018. In this study, there was an increase in prostate, colon, and lung cancers among males for the period. Breast, lung, colon, blood, and ovarian cancers had increased among females for the same period. The study findings determined that prostate cancer has remained the leading cancer in males since 2008, which corroborates previous work [14]. Similar findings were observed by Yang et al. highlighting that the most common cancers among both sexes were colon and lung cancers [14].

It is evident from the outcome that the occurrence of major types of cancer varies across different ethnicities with the highest frequency of occurrence reported among Afro-Trinidadians followed by Indo-Trinidadians. The outcome of the study regarding the high incidence of major types of cancers among individuals from African backgrounds supports the previous evidence [15]. Yedjou et al. [16] observed that breast cancer incidence is high among African-Americans. According to Taitt et al. [17], the incidence of prostate cancer is higher among individuals of African descent. DeSantis et al. [18] also reported that the incidence of lung cancer is high among African-Americans.

### Mortality

The outcome indicates that among males and females the mortality due to major types of cancer such as prostate, lung, breast, and blood cancers declined during this period. Mortality due to breast and lung cancers also declined among females. The findings of this study contradict the observations made by Azamjah et al. [19] which showed an increase in breast cancer mortality. However, Gomez et al. [20] reported observations similar to the study findings which showed a decline in breast cancer mortality among women. Siegel et al. [21] also reported a decline in lung cancer mortality. The study findings suggest that colon cancer mortality increased slightly during 2008–2018 for both males and females. The findings also indicate that ovarian cancer mortality slightly increased during the period 2008–2018.

### Staging

Early diagnosis of cancer is important in the management and treatment [22]. Most females diagnosed with breast cancer are at a localized stage which suggests early diagnosis [23]. However, most males are diagnosed with breast cancer at either a distant or a regional stage, suggesting late diagnosis [23]. Most participants were diagnosed with a distant stage of blood cancer. The majority of staging diagnosis for colon and lung were either distant or unknown for both sexes. Late detection of those cancers can result in poorer prognosis and impact negatively on survival [24, 25]. The outcome therefore suggests that most participants are diagnosed late for leading cancers resulting in the cancer metastasizing in other sites of the body. However, most males are diagnosed with prostate cancer at an unknown stage when the information is not enough to figure out the stage or poor documentation techniques.

## Limitations

There are various limitations that should be taken into consideration in the study. The major limitation is the scope. The study did not assess how the incidence varies across the different age groups due to the lack of national data showing the population per age group. The ability of the findings to inform health policy efforts is limited by the failure to include other parameters such as diagnosis and screening and relating the parameters to the reported incidence and mortality rates.

## Conclusion

The study provides insights into cancer incidence and mortality from 2008 to 2018 in Trinidad and Tobago. The data provided in this study regarding the incidence and mortality of cancer could inform the actions taken by the healthcare providers and the relevant authorities to address public health concerns relating to cancer. Based on this study, there is a need to set healthcare policies to address the expected high incidence of prostate, lung, blood, colon, and pancreatic cancers among males. Steps should also be taken to address the expected high incidence of breast, blood, colon, ovarian, and lung cancers among females. The study outcome also highlights the need to focus on cancer occurrence among Afro-Trinidadians and Indo-Trinidadians. There is also a need to carry out early diagnosis to ensure early staging of breast cancer in males and blood cancer, colon cancer and lung cancer among males and females.

## Supplementary Information

**Additional file 1**: SPSS outputs of cancer mortality in Trinidad and Tobago from 2008 to 2018.

## Data Availability

The data that supports the findings of this study is available from the Trinidad and Tobago Cancer Registry and the TT Central Statistical Office upon request.

## Abbreviations

Afro-Trinidadian: Persons from Trinidad and Tobago who are of African Descent
Indo-Trinidadian: Persons from Trinidad and Tobago who are of Indian Descent
NCRT&T: National Cancer Registry of Trinidad and Tobago
Unknown site: Unknown origin or primary site
