# Supplementary material for: Cancer morbidity and mortality trends in Trinidad and Tobago (2008–2018)
Source: J Health Popul Nutr. 2023 Jun 27;42:58. doi: 10.1186/s41043-023-00395-1 (PMC10303325; doi:10.1186/s41043-023-00395-1)
Supplement: Supplementary file 1 — Additional file 1: SPSS outputs of cancer mortality in Trinidad and Tobago from 2008 to 2018. [file 41043_2023_395_MOESM1_ESM.docx]

SPSS OUTPUTS: Mortality

| **Year * Cause of Death * AGEAGG * Sex Crosstabulation** | | | | | | | |
| --- | --- | --- | --- | --- | --- | --- | --- |
| Count |  |  |  |  |  |  |  |
| Sex |  |  | | | | | Total |
|  |  |  |  |  |  |  |  |
| Female |  |  |  |  |  |  | 1 |
|  | 15-19 |  | 2018 | 3 | 0 |  | 3 |
|  | 25-29 |  | 2018 | 0 | 1 |  | 1 |
|  | 30-34 |  | 2018 | 2 | 0 | 0 | 2 |
|  | 35-39 |  | 2018 | 2 | 0 | 0 | 2 |
|  | 40-44 |  | 2018 | 8 | 2 | 0 | 10 |
|  | 45-49 |  | 2018 | 7 | 4 | 0 | 11 |
|  | 5-9 |  | 2018 | 1 | 0 |  | 1 |
|  | 50-54 |  | 2018 | 12 | 7 | 1 | 20 |
|  | 55-59 |  | 2018 | 26 | 6 | 1 | 33 |
|  | 60-64 |  | 2018 | 29 | 9 | 0 | 38 |
|  | 65-69 |  | 2018 | 26 | 11 | 1 | 38 |
|  | 70-74 |  | 2018 | 27 | 18 | 0 | 45 |
|  | 75-79 |  | 2018 | 24 | 7 | 0 | 31 |
|  | 80-84 |  | 2018 | 23 | 11 | 0 | 34 |
|  | 85 AND OVER |  | 2018 | 26 | 7 | 0 | 33 |
|  |  | Total |  | 249 | 172 | 1 | 422 |
|  | Total | Year | 1993 | 1 | 1 | 0 | 2 |
|  |  |  | 1995 | 0 | 2 | 0 | 2 |
|  |  |  | 1996 | 1 | 0 | 0 | 1 |
|  |  |  | 1998 | 1 | 0 | 0 | 1 |
|  |  |  | 2001 | 2 | 0 | 0 | 2 |
|  |  |  | 2002 | 1 | 0 | 0 | 1 |
|  |  |  | 2003 | 0 | 2 | 0 | 2 |
|  |  |  | 2004 | 2 | 0 | 0 | 2 |
|  |  |  | 2005 | 1 | 0 | 1 | 2 |
|  |  |  | 2006 | 1 | 1 | 0 | 2 |
|  |  |  | 2007 | 2 | 2 | 0 | 4 |
|  |  |  | 2008 | 273 | 100 | 5 | 378 |
|  |  |  | 2009 | 220 | 141 | 6 | 367 |
|  |  |  | 2010 | 158 | 159 | 2 | 319 |
|  |  |  | 2011 | 201 | 195 | 3 | 399 |
|  |  |  | 2012 | 167 | 171 | 4 | 342 |
|  |  |  | 2013 | 162 | 140 | 2 | 304 |
|  |  |  | 2014 | 162 | 154 | 12 | 328 |
|  |  |  | 2015 | 214 | 187 | 9 | 410 |
|  |  |  | 2016 | 174 | 187 | 4 | 365 |
|  |  |  | 2017 | 217 | 164 | 12 | 393 |
|  |  |  | 2018 | 216 | 83 | 3 | 302 |
|  |  | Total |  | 2176 | 1689 | 63 | 3928 |
| Male | 25-29 |  | 2018 | 2 | 0 |  | 2 |
|  | 3 years |  | 2018 | 1 | 1 |  | 2 |
|  | 30-34 |  | 2018 | 4 | 1 |  | 5 |
|  | 35-39 |  | 2018 | 8 | 4 | 0 | 12 |
|  | 40-44 |  | 2018 | 9 | 4 | 1 | 14 |
|  | 45-49 |  | 2018 | 9 | 5 | 1 | 15 |
|  | 50-54 |  | 2018 | 18 | 6 | 2 | 26 |
|  | 55-59 |  | 2018 | 29 | 13 | 0 | 42 |
|  | 60-64 |  | 2018 | 45 | 24 | 5 | 74 |
|  | 65-69 |  | 2018 | 49 | 24 | 3 | 76 |
|  | 70-74 |  | 2018 | 48 | 14 | 1 | 63 |
|  | 75-79 |  | 2018 | 42 | 29 | 1 | 72 |
|  | 80-84 |  | 2018 | 46 | 8 | 1 | 55 |
|  | 85 AND OVER |  | 2018 | 35 | 18 | 1 | 54 |
|  |  | Total |  | 349 | 315 | 5 | 669 |
|  |  | Year | 2012 | 1 |  |  | 1 |
|  | under 1 |  | 2018 | 1 |  |  | 1 |
|  |  | Total |  | 2 |  |  | 2 |
|  | Total | Year | 1998 | 1 | 0 | 0 | 1 |
|  |  |  | 1999 | 1 | 0 | 0 | 1 |
|  |  |  | 2000 | 3 | 1 | 0 | 4 |
|  |  |  | 2001 | 1 | 0 | 0 | 1 |
|  |  |  | 2002 | 3 | 1 | 0 | 4 |
|  |  |  | 2003 | 3 | 2 | 0 | 5 |
|  |  |  | 2004 | 2 | 0 | 0 | 2 |
|  |  |  | 2005 | 4 | 2 | 0 | 6 |
|  |  |  | 2006 | 2 | 1 | 0 | 3 |
|  |  |  | 2007 | 1 | 2 | 0 | 3 |
|  |  |  | 2008 | 402 | 140 | 4 | 546 |
|  |  |  | 2009 | 295 | 236 | 8 | 539 |
|  |  |  | 2010 | 267 | 261 | 2 | 530 |
|  |  |  | 2011 | 259 | 235 | 0 | 494 |
|  |  |  | 2012 | 268 | 259 | 3 | 530 |
|  |  |  | 2013 | 208 | 250 | 4 | 462 |
|  |  |  | 2014 | 220 | 257 | 10 | 487 |
|  |  |  | 2015 | 290 | 275 | 11 | 576 |
|  |  |  | 2016 | 225 | 233 | 14 | 472 |
|  |  |  | 2017 | 299 | 276 | 11 | 586 |
|  |  |  | 2018 | 346 | 151 | 16 | 513 |
|  |  | Total |  | 3100 | 2582 | 83 | 5765 |
| Total |  | Year | 1993 |  | 1 |  | 1 |
|  |  | Total |  |  | 1 |  | 1 |
|  | 1 years | Year | 2009 | 1 | 0 |  | 1 |
|  |  |  | 2010 | 0 | 1 |  | 1 |
|  |  |  | 2011 | 1 | 1 |  | 2 |
|  |  |  | 2012 | 1 | 0 |  | 1 |
|  |  |  | 2016 | 1 | 0 |  | 1 |
|  |  | Total |  | 4 | 2 |  | 6 |
|  | 10-14 | Year | 2008 | 0 | 1 |  | 1 |
|  |  |  | 2009 | 1 | 1 |  | 2 |
|  |  |  | 2010 | 1 | 1 |  | 2 |
|  |  |  | 2011 | 1 | 0 |  | 1 |
|  |  |  | 2013 | 2 | 0 |  | 2 |
|  |  |  | 2015 | 1 | 0 |  | 1 |
|  |  |  | 2017 | 1 | 1 |  | 2 |
|  |  | Total |  | 7 | 4 |  | 11 |
|  | 15-19 | Year | 2008 | 0 | 1 |  | 1 |
|  |  |  | 2009 | 1 | 1 |  | 2 |
|  |  |  | 2010 | 1 | 0 |  | 1 |
|  |  |  | 2011 | 1 | 4 |  | 5 |
|  |  |  | 2012 | 0 | 2 |  | 2 |
|  |  |  | 2013 | 2 | 1 |  | 3 |
|  |  |  | 2014 | 0 | 1 |  | 1 |
|  |  |  | 2015 | 0 | 1 |  | 1 |
|  |  |  | 2017 | 0 | 2 |  | 2 |
|  |  |  | 2018 | 3 | 0 |  | 3 |
|  |  | Total |  | 8 | 13 |  | 21 |
|  | 2 years | Year | 2009 | 1 |  | 0 | 1 |
|  |  |  | 2011 | 1 |  | 0 | 1 |
|  |  |  | 2012 | 0 |  | 1 | 1 |
|  |  | Total |  | 2 |  | 1 | 3 |
|  | 20-24 | Year | 2008 | 3 | 0 |  | 3 |
|  |  |  | 2009 | 3 | 3 |  | 6 |
|  |  |  | 2010 | 1 | 0 |  | 1 |
|  |  |  | 2011 | 2 | 1 |  | 3 |
|  |  |  | 2012 | 2 | 1 |  | 3 |
|  |  |  | 2013 | 1 | 2 |  | 3 |
|  |  |  | 2014 | 1 | 1 |  | 2 |
|  |  |  | 2015 | 1 | 3 |  | 4 |
|  |  |  | 2016 | 1 | 0 |  | 1 |
|  |  |  | 2017 | 2 | 0 |  | 2 |
|  |  | Total |  | 17 | 11 |  | 28 |
|  | 25-29 | Year | 2008 | 2 | 1 |  | 3 |
|  |  |  | 2009 | 3 | 4 |  | 7 |
|  |  |  | 2010 | 2 | 4 |  | 6 |
|  |  |  | 2011 | 6 | 2 |  | 8 |
|  |  |  | 2012 | 3 | 2 |  | 5 |
|  |  |  | 2013 | 2 | 3 |  | 5 |
|  |  |  | 2014 | 2 | 2 |  | 4 |
|  |  |  | 2015 | 3 | 3 |  | 6 |
|  |  |  | 2016 | 1 | 1 |  | 2 |
|  |  |  | 2017 | 0 | 2 |  | 2 |
|  |  |  | 2018 | 2 | 1 |  | 3 |
|  |  | Total |  | 26 | 25 |  | 51 |
|  | 3 years | Year | 2008 | 2 | 0 |  | 2 |
|  |  |  | 2010 | 1 | 0 |  | 1 |
|  |  |  | 2011 | 0 | 1 |  | 1 |
|  |  |  | 2013 | 0 | 1 |  | 1 |
|  |  |  | 2014 | 1 | 0 |  | 1 |
|  |  |  | 2018 | 1 | 1 |  | 2 |
|  |  | Total |  | 5 | 3 |  | 8 |
|  | 30-34 | Year | 2002 | 1 | 0 | 0 | 1 |
|  |  |  | 2008 | 6 | 1 | 0 | 7 |
|  |  |  | 2009 | 5 | 9 | 0 | 14 |
|  |  |  | 2010 | 4 | 6 | 0 | 10 |
|  |  |  | 2011 | 7 | 2 | 0 | 9 |
|  |  |  | 2012 | 3 | 2 | 0 | 5 |
|  |  |  | 2013 | 5 | 4 | 0 | 9 |
|  |  |  | 2014 | 3 | 5 | 0 | 8 |
|  |  |  | 2015 | 4 | 6 | 1 | 11 |
|  |  |  | 2016 | 6 | 1 | 0 | 7 |
|  |  |  | 2017 | 5 | 4 | 0 | 9 |
|  |  |  | 2018 | 6 | 1 | 0 | 7 |
|  |  | Total |  | 55 | 41 | 1 | 97 |
|  | 35-39 | Year | 2002 | 1 | 0 | 0 | 1 |
|  |  |  | 2008 | 18 | 6 | 0 | 24 |
|  |  |  | 2009 | 5 | 3 | 0 | 8 |
|  |  |  | 2010 | 7 | 5 | 1 | 13 |
|  |  |  | 2011 | 5 | 4 | 0 | 9 |
|  |  |  | 2012 | 7 | 8 | 0 | 15 |
|  |  |  | 2013 | 4 | 10 | 0 | 14 |
|  |  |  | 2014 | 6 | 7 | 2 | 15 |
|  |  |  | 2015 | 8 | 8 | 0 | 16 |
|  |  |  | 2016 | 7 | 3 | 0 | 10 |
|  |  |  | 2017 | 11 | 4 | 0 | 15 |
|  |  |  | 2018 | 10 | 4 | 0 | 14 |
|  |  | Total |  | 89 | 62 | 3 | 154 |
|  | 4 years | Year | 2008 | 1 | 0 | 0 | 1 |
|  |  |  | 2011 | 1 | 1 | 0 | 2 |
|  |  |  | 2013 | 0 | 1 | 0 | 1 |
|  |  |  | 2014 | 0 | 0 | 1 | 1 |
|  |  | Total |  | 2 | 2 | 1 | 5 |
|  | 40-44 | Year | 2001 | 1 | 0 | 0 | 1 |
|  |  |  | 2008 | 23 | 11 | 1 | 35 |
|  |  |  | 2009 | 19 | 14 | 0 | 33 |
|  |  |  | 2010 | 15 | 7 | 0 | 22 |
|  |  |  | 2011 | 12 | 16 | 0 | 28 |
|  |  |  | 2012 | 13 | 10 | 0 | 23 |
|  |  |  | 2013 | 9 | 9 | 0 | 18 |
|  |  |  | 2014 | 9 | 11 | 0 | 20 |
|  |  |  | 2015 | 10 | 6 | 0 | 16 |
|  |  |  | 2016 | 10 | 9 | 0 | 19 |
|  |  |  | 2017 | 11 | 11 | 0 | 22 |
|  |  |  | 2018 | 17 | 6 | 1 | 24 |
|  |  | Total |  | 149 | 110 | 2 | 261 |
|  | 45-49 | Year | 2003 | 0 | 1 | 0 | 1 |
|  |  |  | 2004 | 1 | 0 | 0 | 1 |
|  |  |  | 2005 | 0 | 1 | 0 | 1 |
|  |  |  | 2007 | 1 | 0 | 0 | 1 |
|  |  |  | 2008 | 25 | 19 | 1 | 45 |
|  |  |  | 2009 | 35 | 19 | 1 | 55 |
|  |  |  | 2010 | 18 | 24 | 0 | 42 |
|  |  |  | 2011 | 23 | 10 | 0 | 33 |
|  |  |  | 2012 | 16 | 18 | 0 | 34 |
|  |  |  | 2013 | 12 | 12 | 0 | 24 |
|  |  |  | 2014 | 19 | 13 | 1 | 33 |
|  |  |  | 2015 | 15 | 14 | 2 | 31 |
|  |  |  | 2016 | 16 | 18 | 0 | 34 |
|  |  |  | 2017 | 22 | 14 | 1 | 37 |
|  |  |  | 2018 | 16 | 9 | 1 | 26 |
|  |  | Total |  | 219 | 172 | 7 | 398 |
|  | 5-9 | Year | 2009 | 0 | 4 |  | 4 |
|  |  |  | 2011 | 2 | 1 |  | 3 |
|  |  |  | 2012 | 0 | 4 |  | 4 |
|  |  |  | 2013 | 0 | 1 |  | 1 |
|  |  |  | 2014 | 2 | 2 |  | 4 |
|  |  |  | 2015 | 0 | 1 |  | 1 |
|  |  |  | 2016 | 0 | 1 |  | 1 |
|  |  |  | 2017 | 0 | 1 |  | 1 |
|  |  |  | 2018 | 1 | 0 |  | 1 |
|  |  | Total |  | 5 | 15 |  | 20 |
|  | 50-54 | Year | 2002 | 0 | 1 | 0 | 1 |
|  |  |  | 2006 | 0 | 1 | 0 | 1 |
|  |  |  | 2008 | 64 | 24 | 1 | 89 |
|  |  |  | 2009 | 39 | 25 | 1 | 65 |
|  |  |  | 2010 | 30 | 34 | 1 | 65 |
|  |  |  | 2011 | 35 | 28 | 1 | 64 |
|  |  |  | 2012 | 40 | 32 | 0 | 72 |
|  |  |  | 2013 | 28 | 29 | 1 | 58 |
|  |  |  | 2014 | 24 | 22 | 2 | 48 |
|  |  |  | 2015 | 37 | 31 | 2 | 70 |
|  |  |  | 2016 | 19 | 28 | 2 | 49 |
|  |  |  | 2017 | 27 | 31 | 2 | 60 |
|  |  |  | 2018 | 30 | 13 | 3 | 46 |
|  |  | Total |  | 373 | 299 | 16 | 688 |
|  | 55-59 | Year | 1996 | 1 | 0 | 0 | 1 |
|  |  |  | 2003 | 1 | 1 | 0 | 2 |
|  |  |  | 2005 | 2 | 0 | 0 | 2 |
|  |  |  | 2006 | 1 | 0 | 0 | 1 |
|  |  |  | 2008 | 49 | 21 | 3 | 73 |
|  |  |  | 2009 | 58 | 28 | 0 | 86 |
|  |  |  | 2010 | 35 | 40 | 0 | 75 |
|  |  |  | 2011 | 54 | 53 | 1 | 108 |
|  |  |  | 2012 | 47 | 40 | 0 | 87 |
|  |  |  | 2013 | 38 | 34 | 0 | 72 |
|  |  |  | 2014 | 35 | 53 | 2 | 90 |
|  |  |  | 2015 | 46 | 45 | 1 | 92 |
|  |  |  | 2016 | 50 | 46 | 1 | 97 |
|  |  |  | 2017 | 46 | 35 | 3 | 84 |
|  |  |  | 2018 | 55 | 19 | 1 | 75 |
|  |  | Total |  | 518 | 415 | 12 | 945 |
|  | 60-64 | Year | 1995 | 0 | 2 | 0 | 2 |
|  |  |  | 2003 | 1 | 1 | 0 | 2 |
|  |  |  | 2004 | 1 | 0 | 0 | 1 |
|  |  |  | 2005 | 2 | 0 | 0 | 2 |
|  |  |  | 2006 | 1 | 0 | 0 | 1 |
|  |  |  | 2008 | 77 | 33 | 0 | 110 |
|  |  |  | 2009 | 58 | 55 | 0 | 113 |
|  |  |  | 2010 | 51 | 40 | 0 | 91 |
|  |  |  | 2011 | 49 | 57 | 0 | 106 |
|  |  |  | 2012 | 30 | 62 | 1 | 93 |
|  |  |  | 2013 | 52 | 33 | 2 | 87 |
|  |  |  | 2014 | 44 | 39 | 3 | 86 |
|  |  |  | 2015 | 64 | 53 | 5 | 122 |
|  |  |  | 2016 | 50 | 54 | 4 | 108 |
|  |  |  | 2017 | 80 | 59 | 1 | 140 |
|  |  |  | 2018 | 74 | 33 | 5 | 112 |
|  |  | Total |  | 634 | 521 | 21 | 1176 |
|  | 65-69 | Year | 1993 | 1 | 0 | 0 | 1 |
|  |  |  | 2000 | 1 | 1 | 0 | 2 |
|  |  |  | 2003 | 0 | 1 | 0 | 1 |
|  |  |  | 2006 | 1 | 1 | 0 | 2 |
|  |  |  | 2007 | 0 | 2 | 0 | 2 |
|  |  |  | 2008 | 77 | 29 | 1 | 107 |
|  |  |  | 2009 | 74 | 40 | 4 | 118 |
|  |  |  | 2010 | 47 | 47 | 0 | 94 |
|  |  |  | 2011 | 63 | 59 | 0 | 122 |
|  |  |  | 2012 | 57 | 46 | 1 | 104 |
|  |  |  | 2013 | 51 | 58 | 1 | 110 |
|  |  |  | 2014 | 68 | 65 | 5 | 138 |
|  |  |  | 2015 | 62 | 66 | 1 | 129 |
|  |  |  | 2016 | 64 | 64 | 3 | 131 |
|  |  |  | 2017 | 63 | 47 | 2 | 112 |
|  |  |  | 2018 | 75 | 35 | 4 | 114 |
|  |  | Total |  | 704 | 561 | 22 | 1287 |
|  | 70-74 | Year | 2000 | 2 | 0 | 0 | 2 |
|  |  |  | 2004 | 1 | 0 | 0 | 1 |
|  |  |  | 2005 | 1 | 1 | 1 | 3 |
|  |  |  | 2007 | 1 | 1 | 0 | 2 |
|  |  |  | 2008 | 89 | 20 | 1 | 110 |
|  |  |  | 2009 | 66 | 37 | 2 | 105 |
|  |  |  | 2010 | 65 | 59 | 0 | 124 |
|  |  |  | 2011 | 55 | 53 | 0 | 108 |
|  |  |  | 2012 | 67 | 47 | 0 | 114 |
|  |  |  | 2013 | 42 | 60 | 0 | 102 |
|  |  |  | 2014 | 52 | 38 | 2 | 92 |
|  |  |  | 2015 | 91 | 69 | 1 | 161 |
|  |  |  | 2016 | 62 | 50 | 5 | 117 |
|  |  |  | 2017 | 78 | 79 | 3 | 160 |
|  |  |  | 2018 | 75 | 32 | 1 | 108 |
|  |  | Total |  | 747 | 546 | 16 | 1309 |
|  | 75-79 | Year | 1998 | 2 | 0 | 0 | 2 |
|  |  |  | 1999 | 1 | 0 | 0 | 1 |
|  |  |  | 2001 | 1 | 0 | 0 | 1 |
|  |  |  | 2003 | 1 | 0 | 0 | 1 |
|  |  |  | 2004 | 1 | 0 | 0 | 1 |
|  |  |  | 2007 | 0 | 1 | 0 | 1 |
|  |  |  | 2008 | 84 | 26 | 1 | 111 |
|  |  |  | 2009 | 48 | 57 | 2 | 107 |
|  |  |  | 2010 | 49 | 55 | 0 | 104 |
|  |  |  | 2011 | 43 | 49 | 1 | 93 |
|  |  |  | 2012 | 56 | 60 | 4 | 120 |
|  |  |  | 2013 | 45 | 34 | 1 | 80 |
|  |  |  | 2014 | 45 | 50 | 4 | 99 |
|  |  |  | 2015 | 49 | 57 | 5 | 111 |
|  |  |  | 2016 | 34 | 48 | 2 | 84 |
|  |  |  | 2017 | 63 | 50 | 5 | 118 |
|  |  |  | 2018 | 66 | 36 | 1 | 103 |
|  |  | Total |  | 588 | 523 | 26 | 1137 |
|  | 80-84 | Year | 2001 | 1 | 0 | 0 | 1 |
|  |  |  | 2002 | 2 | 0 | 0 | 2 |
|  |  |  | 2007 | 1 | 0 | 0 | 1 |
|  |  |  | 2008 | 80 | 15 | 0 | 95 |
|  |  |  | 2009 | 41 | 31 | 3 | 75 |
|  |  |  | 2010 | 38 | 47 | 2 | 87 |
|  |  |  | 2011 | 44 | 42 | 0 | 86 |
|  |  |  | 2012 | 40 | 43 | 0 | 83 |
|  |  |  | 2013 | 29 | 50 | 1 | 80 |
|  |  |  | 2014 | 37 | 56 | 0 | 93 |
|  |  |  | 2015 | 56 | 43 | 1 | 100 |
|  |  |  | 2016 | 41 | 56 | 1 | 98 |
|  |  |  | 2017 | 45 | 56 | 3 | 104 |
|  |  |  | 2018 | 69 | 19 | 1 | 89 |
|  |  | Total |  | 524 | 458 | 12 | 994 |
|  | 85 AND OVER | Year | 2008 | 75 | 32 | 0 | 107 |
|  |  |  | 2009 | 57 | 46 | 1 | 104 |
|  |  |  | 2010 | 60 | 50 | 0 | 110 |
|  |  |  | 2011 | 55 | 46 | 0 | 101 |
|  |  |  | 2012 | 52 | 53 | 0 | 105 |
|  |  |  | 2013 | 48 | 48 | 0 | 96 |
|  |  |  | 2014 | 34 | 46 | 0 | 80 |
|  |  |  | 2015 | 57 | 56 | 1 | 114 |
|  |  |  | 2016 | 37 | 41 | 0 | 78 |
|  |  |  | 2017 | 62 | 44 | 3 | 109 |
|  |  |  | 2018 | 61 | 25 | 1 | 87 |
|  |  | Total |  | 598 | 487 | 6 | 1091 |
|  | under 1 | Year | 2012 | 1 |  |  | 1 |
|  |  |  | 2018 | 1 |  |  | 1 |
|  |  | Total |  | 2 |  |  | 2 |
|  | Total | Year | 1993 | 1 | 1 | 0 | 2 |
|  |  |  | 1995 | 0 | 2 | 0 | 2 |
|  |  |  | 1996 | 1 | 0 | 0 | 1 |
|  |  |  | 1998 | 2 | 0 | 0 | 2 |
|  |  |  | 1999 | 1 | 0 | 0 | 1 |
|  |  |  | 2000 | 3 | 1 | 0 | 4 |
|  |  |  | 2001 | 3 | 0 | 0 | 3 |
|  |  |  | 2002 | 4 | 1 | 0 | 5 |
|  |  |  | 2003 | 3 | 4 | 0 | 7 |
|  |  |  | 2004 | 4 | 0 | 0 | 4 |
|  |  |  | 2005 | 5 | 2 | 1 | 8 |
|  |  |  | 2006 | 3 | 2 | 0 | 5 |
|  |  |  | 2007 | 3 | 4 | 0 | 7 |
|  |  |  | 2008 | 675 | 240 | 9 | 924 |
|  |  |  | 2009 | 515 | 377 | 14 | 906 |
|  |  |  | 2010 | 425 | 420 | 4 | 849 |
|  |  |  | 2011 | 460 | 430 | 3 | 893 |
|  |  |  | 2012 | 435 | 430 | 7 | 872 |
|  |  |  | 2013 | 370 | 390 | 6 | 766 |
|  |  |  | 2014 | 382 | 411 | 22 | 815 |
|  |  |  | 2015 | 504 | 462 | 20 | 986 |
|  |  |  | 2016 | 399 | 420 | 18 | 837 |
|  |  |  | 2017 | 516 | 440 | 23 | 979 |
|  |  |  | 2018 | 562 | 234 | 19 | 815 |
|  |  | Total |  | 5276 | 4271 | 146 | 9693 |

SPSS OUTPUTS: Incidence

| **Year * Topography- Site * Sex Crosstabulation** | | | | | | | | | | | | | |
| --- | --- | --- | --- | --- | --- | --- | --- | --- | --- | --- | --- | --- | --- |
| Count |  |  |  |  |  |  |  |  |  |  |  |  |  |
| Sex |  |  | Topography- Site |  |  |  |  |  |  |  |  |  | Total |
|  |  |  | Anus, Anal Canal | Blood, Spleen | Breast | Bronchus, Lung | Colon | Ovary | Pancreas | Prostate | Rectum | Stomach |  |
| Female | Year | 1993 | 0 | 0 | 2 | 0 | 0 | 0 | 0 |  | 0 | 0 | 2 |
|  |  | 1995 | 0 | 0 | 1 | 0 | 1 | 0 | 0 |  | 0 | 0 | 2 |
|  |  | 1996 | 0 | 0 | 1 | 0 | 0 | 0 | 0 |  | 0 | 0 | 1 |
|  |  | 1998 | 0 | 1 | 0 | 0 | 0 | 0 | 0 |  | 0 | 0 | 1 |
|  |  | 2001 | 0 | 0 | 1 | 0 | 1 | 0 | 0 |  | 0 | 0 | 2 |
|  |  | 2002 | 0 | 0 | 1 | 0 | 0 | 0 | 0 |  | 0 | 0 | 1 |
|  |  | 2003 | 0 | 0 | 2 | 0 | 0 | 0 | 0 |  | 0 | 0 | 2 |
|  |  | 2004 | 0 | 0 | 2 | 0 | 0 | 0 | 0 |  | 0 | 0 | 2 |
|  |  | 2005 | 0 | 0 | 2 | 0 | 0 | 0 | 0 |  | 0 | 0 | 2 |
|  |  | 2006 | 0 | 0 | 0 | 0 | 1 | 1 | 0 |  | 0 | 0 | 2 |
|  |  | 2007 | 0 | 0 | 4 | 0 | 0 | 0 | 0 |  | 0 | 0 | 4 |
|  |  | 2008 | 3 | 35 | 385 | 35 | 74 | 67 | 34 |  | 27 | 25 | 685 |
|  |  | 2009 | 4 | 38 | 381 | 43 | 64 | 58 | 32 |  | 23 | 28 | 671 |
|  |  | 2010 | 2 | 37 | 330 | 38 | 62 | 66 | 32 |  | 22 | 11 | 600 |
|  |  | 2011 | 4 | 48 | 368 | 48 | 77 | 69 | 33 |  | 21 | 26 | 694 |
|  |  | 2012 | 5 | 45 | 249 | 34 | 56 | 53 | 24 |  | 15 | 23 | 504 |
|  |  | 2013 | 4 | 41 | 230 | 32 | 60 | 47 | 29 |  | 12 | 21 | 476 |
|  |  | 2014 | 1 | 38 | 276 | 44 | 58 | 60 | 31 |  | 13 | 22 | 543 |
|  |  | 2015 | 3 | 55 | 338 | 49 | 78 | 63 | 38 |  | 23 | 22 | 669 |
|  |  | 2016 | 5 | 52 | 313 | 49 | 68 | 72 | 32 |  | 21 | 22 | 634 |
|  |  | 2017 | 3 | 41 | 364 | 46 | 62 | 83 | 38 |  | 16 | 17 | 670 |
|  |  | 2018 | 7 | 53 | 435 | 49 | 85 | 81 | 35 |  | 15 | 20 | 780 |
|  | Total |  | 41 | 484 | 3685 | 467 | 747 | 720 | 358 |  | 208 | 237 | 6947 |
| Male | Year | 1998 | 0 | 0 | 0 | 0 | 0 |  | 0 | 1 | 0 | 0 | 1 |
|  |  | 1999 | 0 | 0 | 0 | 0 | 0 |  | 0 | 1 | 0 | 0 | 1 |
|  |  | 2000 | 0 | 0 | 0 | 0 | 0 |  | 0 | 4 | 0 | 0 | 4 |
|  |  | 2001 | 0 | 0 | 0 | 0 | 0 |  | 0 | 1 | 0 | 0 | 1 |
|  |  | 2002 | 0 | 1 | 0 | 0 | 0 |  | 0 | 3 | 0 | 0 | 4 |
|  |  | 2003 | 0 | 1 | 1 | 0 | 0 |  | 0 | 2 | 1 | 0 | 5 |
|  |  | 2004 | 0 | 0 | 0 | 0 | 0 |  | 0 | 2 | 0 | 0 | 2 |
|  |  | 2005 | 0 | 0 | 0 | 0 | 1 |  | 0 | 5 | 0 | 0 | 6 |
|  |  | 2006 | 0 | 0 | 0 | 0 | 0 |  | 0 | 3 | 0 | 0 | 3 |
|  |  | 2007 | 0 | 0 | 0 | 0 | 0 |  | 0 | 3 | 0 | 0 | 3 |
|  |  | 2008 | 7 | 51 | 4 | 117 | 83 |  | 44 | 393 | 35 | 40 | 774 |
|  |  | 2009 | 3 | 48 | 8 | 134 | 77 |  | 52 | 379 | 31 | 33 | 765 |
|  |  | 2010 | 3 | 45 | 3 | 104 | 74 |  | 33 | 400 | 27 | 36 | 725 |
|  |  | 2011 | 5 | 43 | 4 | 112 | 74 |  | 32 | 344 | 28 | 30 | 672 |
|  |  | 2012 | 1 | 56 | 3 | 100 | 63 |  | 39 | 351 | 24 | 33 | 670 |
|  |  | 2013 | 2 | 49 | 8 | 117 | 51 |  | 37 | 305 | 14 | 25 | 608 |
|  |  | 2014 | 4 | 62 | 4 | 128 | 61 |  | 32 | 314 | 30 | 18 | 653 |
|  |  | 2015 | 6 | 76 | 6 | 139 | 79 |  | 49 | 380 | 24 | 25 | 784 |
|  |  | 2016 | 3 | 60 | 6 | 107 | 77 |  | 36 | 341 | 31 | 27 | 688 |
|  |  | 2017 | 3 | 67 | 7 | 149 | 63 |  | 47 | 406 | 25 | 22 | 789 |
|  |  | 2018 | 3 | 47 | 9 | 163 | 92 |  | 48 | 484 | 48 | 30 | 924 |
|  | Total |  | 40 | 606 | 63 | 1370 | 795 |  | 449 | 4122 | 318 | 319 | 8082 |
| Total | Year | 1993 | 0 | 0 | 2 | 0 | 0 | 0 | 0 | 0 | 0 | 0 | 2 |
|  |  | 1995 | 0 | 0 | 1 | 0 | 1 | 0 | 0 | 0 | 0 | 0 | 2 |
|  |  | 1996 | 0 | 0 | 1 | 0 | 0 | 0 | 0 | 0 | 0 | 0 | 1 |
|  |  | 1998 | 0 | 1 | 0 | 0 | 0 | 0 | 0 | 1 | 0 | 0 | 2 |
|  |  | 1999 | 0 | 0 | 0 | 0 | 0 | 0 | 0 | 1 | 0 | 0 | 1 |
|  |  | 2000 | 0 | 0 | 0 | 0 | 0 | 0 | 0 | 4 | 0 | 0 | 4 |
|  |  | 2001 | 0 | 0 | 1 | 0 | 1 | 0 | 0 | 1 | 0 | 0 | 3 |
|  |  | 2002 | 0 | 1 | 1 | 0 | 0 | 0 | 0 | 3 | 0 | 0 | 5 |
|  |  | 2003 | 0 | 1 | 3 | 0 | 0 | 0 | 0 | 2 | 1 | 0 | 7 |
|  |  | 2004 | 0 | 0 | 2 | 0 | 0 | 0 | 0 | 2 | 0 | 0 | 4 |
|  |  | 2005 | 0 | 0 | 2 | 0 | 1 | 0 | 0 | 5 | 0 | 0 | 8 |
|  |  | 2006 | 0 | 0 | 0 | 0 | 1 | 1 | 0 | 3 | 0 | 0 | 5 |
|  |  | 2007 | 0 | 0 | 4 | 0 | 0 | 0 | 0 | 3 | 0 | 0 | 7 |
|  |  | 2008 | 10 | 86 | 389 | 152 | 157 | 67 | 78 | 393 | 62 | 65 | 1459 |
|  |  | 2009 | 7 | 86 | 389 | 177 | 141 | 58 | 84 | 379 | 54 | 61 | 1436 |
|  |  | 2010 | 5 | 82 | 333 | 142 | 136 | 66 | 65 | 400 | 49 | 47 | 1325 |
|  |  | 2011 | 9 | 91 | 372 | 160 | 151 | 69 | 65 | 344 | 49 | 56 | 1366 |
|  |  | 2012 | 6 | 101 | 252 | 134 | 119 | 53 | 63 | 351 | 39 | 56 | 1174 |
|  |  | 2013 | 6 | 90 | 238 | 149 | 111 | 47 | 66 | 305 | 26 | 46 | 1084 |
|  |  | 2014 | 5 | 100 | 280 | 172 | 119 | 60 | 63 | 314 | 43 | 40 | 1196 |
|  |  | 2015 | 9 | 131 | 344 | 188 | 157 | 63 | 87 | 380 | 47 | 47 | 1453 |
|  |  | 2016 | 8 | 112 | 319 | 156 | 145 | 72 | 68 | 341 | 52 | 49 | 1322 |
|  |  | 2017 | 6 | 108 | 371 | 195 | 125 | 83 | 85 | 406 | 41 | 39 | 1459 |
|  |  | 2018 | 10 | 100 | 444 | 212 | 177 | 81 | 83 | 484 | 63 | 50 | 1704 |
|  | Total |  | 81 | 1090 | 3748 | 1837 | 1542 | 720 | 807 | 4122 | 526 | 556 | 15029 |

Ethnicity and incidence

| **Year * Topography- Site * Ethnicity Crosstabulation** | | | | | | | | | | | | | |
| --- | --- | --- | --- | --- | --- | --- | --- | --- | --- | --- | --- | --- | --- |
| Count |  |  |  |  |  |  |  |  |  |  |  |  |  |
| Ethnicity |  |  | Topography- Site |  |  |  |  |  |  |  |  |  | Total |
|  |  |  | Anus, Anal Canal | Blood, Spleen | Breast | Bronchus, Lung | Colon | Ovary | Pancreas | Prostate | Rectum | Stomach |  |
| African | Year | 1993 | 0 | 0 | 1 | 0 | 0 | 0 | 0 | 0 | 0 | 0 | 1 |
|  |  | 1995 | 0 | 0 | 1 | 0 | 0 | 0 | 0 | 0 | 0 | 0 | 1 |
|  |  | 1998 | 0 | 1 | 0 | 0 | 0 | 0 | 0 | 1 | 0 | 0 | 2 |
|  |  | 2000 | 0 | 0 | 0 | 0 | 0 | 0 | 0 | 4 | 0 | 0 | 4 |
|  |  | 2002 | 0 | 1 | 1 | 0 | 0 | 0 | 0 | 2 | 0 | 0 | 4 |
|  |  | 2003 | 0 | 0 | 3 | 0 | 0 | 0 | 0 | 0 | 0 | 0 | 3 |
|  |  | 2004 | 0 | 0 | 0 | 0 | 0 | 0 | 0 | 2 | 0 | 0 | 2 |
|  |  | 2005 | 0 | 0 | 1 | 0 | 1 | 0 | 0 | 4 | 0 | 0 | 6 |
|  |  | 2006 | 0 | 0 | 0 | 0 | 0 | 0 | 0 | 2 | 0 | 0 | 2 |
|  |  | 2007 | 0 | 0 | 2 | 0 | 0 | 0 | 0 | 2 | 0 | 0 | 4 |
|  |  | 2008 | 5 | 25 | 141 | 62 | 56 | 21 | 24 | 180 | 14 | 19 | 547 |
|  |  | 2009 | 5 | 27 | 141 | 61 | 45 | 20 | 32 | 179 | 20 | 21 | 551 |
|  |  | 2010 | 2 | 17 | 121 | 46 | 46 | 20 | 16 | 183 | 15 | 13 | 479 |
|  |  | 2011 | 5 | 19 | 120 | 53 | 54 | 13 | 17 | 150 | 16 | 20 | 467 |
|  |  | 2012 | 1 | 27 | 86 | 35 | 43 | 14 | 23 | 150 | 11 | 15 | 405 |
|  |  | 2013 | 3 | 22 | 73 | 51 | 39 | 12 | 28 | 134 | 10 | 11 | 383 |
|  |  | 2014 | 3 | 34 | 100 | 48 | 26 | 17 | 18 | 137 | 13 | 15 | 411 |
|  |  | 2015 | 7 | 27 | 114 | 53 | 39 | 12 | 29 | 170 | 12 | 11 | 474 |
|  |  | 2016 | 5 | 33 | 119 | 50 | 43 | 23 | 23 | 166 | 15 | 17 | 494 |
|  |  | 2017 | 1 | 27 | 121 | 52 | 42 | 29 | 25 | 194 | 11 | 16 | 518 |
|  |  | 2018 | 2 | 24 | 152 | 65 | 48 | 32 | 34 | 189 | 15 | 17 | 578 |
|  | Total |  | 39 | 284 | 1297 | 576 | 482 | 213 | 269 | 1849 | 152 | 175 | 5336 |
| Indian | Year | 2001 | 0 | 0 | 1 | 0 | 1 | 0 | 0 | 0 | 0 | 0 | 2 |
|  |  | 2003 | 0 | 1 | 0 | 0 | 0 | 0 | 0 | 0 | 0 | 0 | 1 |
|  |  | 2004 | 0 | 0 | 2 | 0 | 0 | 0 | 0 | 0 | 0 | 0 | 2 |
|  |  | 2006 | 0 | 0 | 0 | 0 | 1 | 1 | 0 | 0 | 0 | 0 | 2 |
|  |  | 2007 | 0 | 0 | 0 | 0 | 0 | 0 | 0 | 1 | 0 | 0 | 1 |
|  |  | 2008 | 2 | 18 | 127 | 23 | 38 | 27 | 10 | 47 | 24 | 10 | 326 |
|  |  | 2009 | 1 | 13 | 140 | 37 | 32 | 15 | 15 | 33 | 24 | 16 | 326 |
|  |  | 2010 | 0 | 19 | 114 | 38 | 25 | 23 | 10 | 35 | 19 | 8 | 291 |
|  |  | 2011 | 1 | 19 | 133 | 44 | 41 | 18 | 9 | 38 | 22 | 7 | 332 |
|  |  | 2012 | 0 | 19 | 52 | 25 | 19 | 13 | 8 | 29 | 11 | 10 | 186 |
|  |  | 2013 | 0 | 19 | 57 | 27 | 11 | 7 | 6 | 27 | 9 | 11 | 174 |
|  |  | 2014 | 0 | 20 | 80 | 40 | 24 | 11 | 9 | 43 | 15 | 4 | 246 |
|  |  | 2015 | 0 | 21 | 107 | 42 | 40 | 12 | 11 | 49 | 16 | 11 | 309 |
|  |  | 2016 | 1 | 32 | 92 | 47 | 43 | 17 | 10 | 31 | 18 | 8 | 299 |
|  |  | 2017 | 3 | 24 | 110 | 38 | 31 | 24 | 22 | 41 | 16 | 6 | 315 |
|  |  | 2018 | 2 | 22 | 140 | 46 | 41 | 21 | 19 | 57 | 23 | 11 | 382 |
|  | Total |  | 10 | 227 | 1155 | 407 | 347 | 189 | 129 | 431 | 197 | 102 | 3194 |
| Chinese | Year | 2008 |  | 1 | 1 | 0 | 0 | 0 | 0 | 1 | 0 | 0 | 3 |
|  |  | 2009 |  | 0 | 5 | 3 | 1 | 0 | 1 | 0 | 1 | 0 | 11 |
|  |  | 2010 |  | 1 | 1 | 0 | 0 | 0 | 0 | 3 | 0 | 0 | 5 |
|  |  | 2011 |  | 0 | 3 | 0 | 1 | 0 | 0 | 2 | 0 | 0 | 6 |
|  |  | 2012 |  | 1 | 0 | 0 | 1 | 0 | 0 | 0 | 0 | 0 | 2 |
|  |  | 2013 |  | 0 | 0 | 0 | 0 | 0 | 0 | 1 | 0 | 0 | 1 |
|  |  | 2014 |  | 0 | 0 | 1 | 0 | 0 | 1 | 0 | 0 | 0 | 2 |
|  |  | 2015 |  | 0 | 1 | 2 | 1 | 0 | 0 | 2 | 0 | 0 | 6 |
|  |  | 2016 |  | 1 | 1 | 2 | 1 | 0 | 0 | 1 | 1 | 2 | 9 |
|  |  | 2017 |  | 0 | 1 | 0 | 0 | 0 | 0 | 3 | 0 | 0 | 4 |
|  |  | 2018 |  | 0 | 2 | 1 | 1 | 1 | 0 | 2 | 0 | 0 | 7 |
|  | Total |  |  | 4 | 15 | 9 | 6 | 1 | 2 | 15 | 2 | 2 | 56 |
| Syrian/Lebanese | Year | 2008 |  | 0 | 1 |  | 1 | 0 |  | 0 | 1 |  | 3 |
|  |  | 2009 |  | 0 | 2 |  | 0 | 1 |  | 1 | 0 |  | 4 |
|  |  | 2011 |  | 0 | 1 |  | 0 | 0 |  | 0 | 0 |  | 1 |
|  |  | 2012 |  | 0 | 0 |  | 0 | 0 |  | 1 | 0 |  | 1 |
|  |  | 2013 |  | 1 | 0 |  | 0 | 0 |  | 0 | 0 |  | 1 |
|  |  | 2017 |  | 1 | 0 |  | 0 | 0 |  | 0 | 0 |  | 1 |
|  |  | 2018 |  | 0 | 0 |  | 1 | 0 |  | 0 | 0 |  | 1 |
|  | Total |  |  | 2 | 4 |  | 2 | 1 |  | 2 | 1 |  | 12 |
| White/Caucasian | Year | 2003 |  | 0 | 0 | 0 | 0 | 0 | 0 | 1 | 0 | 0 | 1 |
|  |  | 2008 |  | 0 | 0 | 0 | 2 | 0 | 0 | 6 | 0 | 0 | 8 |
|  |  | 2009 |  | 1 | 3 | 3 | 0 | 0 | 0 | 1 | 0 | 0 | 8 |
|  |  | 2010 |  | 1 | 3 | 3 | 6 | 0 | 1 | 2 | 1 | 0 | 17 |
|  |  | 2011 |  | 2 | 1 | 0 | 0 | 2 | 2 | 0 | 0 | 0 | 7 |
|  |  | 2012 |  | 0 | 3 | 2 | 3 | 0 | 0 | 3 | 0 | 0 | 11 |
|  |  | 2013 |  | 0 | 4 | 3 | 1 | 0 | 0 | 4 | 0 | 0 | 12 |
|  |  | 2014 |  | 0 | 1 | 2 | 0 | 0 | 0 | 1 | 0 | 1 | 5 |
|  |  | 2015 |  | 0 | 3 | 3 | 1 | 0 | 0 | 5 | 1 | 0 | 13 |
|  |  | 2016 |  | 0 | 3 | 2 | 0 | 0 | 1 | 4 | 0 | 0 | 10 |
|  |  | 2017 |  | 0 | 5 | 4 | 2 | 2 | 0 | 3 | 0 | 0 | 16 |
|  |  | 2018 |  | 0 | 2 | 1 | 1 | 1 | 0 | 1 | 0 | 0 | 6 |
|  | Total |  |  | 4 | 28 | 23 | 16 | 5 | 4 | 31 | 2 | 1 | 114 |
| Mixed | Year | 1993 | 0 | 0 | 1 | 0 | 0 | 0 | 0 | 0 | 0 | 0 | 1 |
|  |  | 1996 | 0 | 0 | 1 | 0 | 0 | 0 | 0 | 0 | 0 | 0 | 1 |
|  |  | 2001 | 0 | 0 | 0 | 0 | 0 | 0 | 0 | 1 | 0 | 0 | 1 |
|  |  | 2002 | 0 | 0 | 0 | 0 | 0 | 0 | 0 | 1 | 0 | 0 | 1 |
|  |  | 2003 | 0 | 0 | 0 | 0 | 0 | 0 | 0 | 0 | 1 | 0 | 1 |
|  |  | 2005 | 0 | 0 | 1 | 0 | 0 | 0 | 0 | 1 | 0 | 0 | 2 |
|  |  | 2006 | 0 | 0 | 0 | 0 | 0 | 0 | 0 | 1 | 0 | 0 | 1 |
|  |  | 2007 | 0 | 0 | 2 | 0 | 0 | 0 | 0 | 0 | 0 | 0 | 2 |
|  |  | 2008 | 1 | 12 | 60 | 28 | 14 | 6 | 8 | 37 | 10 | 9 | 185 |
|  |  | 2009 | 0 | 9 | 58 | 25 | 25 | 6 | 9 | 41 | 6 | 5 | 184 |
|  |  | 2010 | 1 | 10 | 57 | 15 | 11 | 4 | 9 | 47 | 9 | 8 | 171 |
|  |  | 2011 | 2 | 9 | 52 | 21 | 19 | 12 | 7 | 53 | 8 | 5 | 188 |
|  |  | 2012 | 4 | 7 | 41 | 18 | 14 | 10 | 8 | 43 | 4 | 3 | 152 |
|  |  | 2013 | 2 | 6 | 29 | 17 | 11 | 4 | 3 | 34 | 1 | 3 | 110 |
|  |  | 2014 | 0 | 5 | 36 | 12 | 15 | 8 | 6 | 36 | 4 | 1 | 123 |
|  |  | 2015 | 2 | 15 | 49 | 27 | 18 | 10 | 5 | 34 | 8 | 3 | 171 |
|  |  | 2016 | 0 | 12 | 51 | 18 | 15 | 7 | 7 | 51 | 11 | 7 | 179 |
|  |  | 2017 | 2 | 15 | 54 | 30 | 10 | 2 | 14 | 35 | 3 | 6 | 171 |
|  |  | 2018 | 5 | 20 | 64 | 18 | 19 | 7 | 3 | 43 | 12 | 7 | 198 |
|  | Total |  | 19 | 120 | 556 | 229 | 171 | 76 | 79 | 458 | 77 | 57 | 1842 |
| Other | Year | 2008 |  | 7 | 10 | 11 | 7 | 4 | 11 | 22 | 2 | 8 | 82 |
|  |  | 2009 |  | 0 | 0 | 0 | 0 | 1 | 0 | 0 | 0 | 0 | 1 |
|  |  | 2010 |  | 0 | 0 | 0 | 0 | 0 | 1 | 0 | 0 | 0 | 1 |
|  |  | 2011 |  | 0 | 0 | 1 | 1 | 0 | 0 | 0 | 0 | 0 | 2 |
|  |  | 2012 |  | 1 | 1 | 0 | 0 | 0 | 0 | 2 | 0 | 0 | 4 |
|  |  | 2014 |  | 0 | 1 | 0 | 0 | 0 | 0 | 0 | 0 | 0 | 1 |
|  |  | 2015 |  | 0 | 1 | 0 | 2 | 0 | 0 | 1 | 0 | 0 | 4 |
|  |  | 2016 |  | 0 | 0 | 1 | 0 | 0 | 0 | 1 | 0 | 0 | 2 |
|  | Total |  |  | 8 | 13 | 13 | 10 | 5 | 12 | 26 | 2 | 8 | 97 |
| Unknown | Year | 1995 | 0 | 0 | 0 | 0 | 1 | 0 | 0 | 0 | 0 | 0 | 1 |
|  |  | 1999 | 0 | 0 | 0 | 0 | 0 | 0 | 0 | 1 | 0 | 0 | 1 |
|  |  | 2003 | 0 | 0 | 0 | 0 | 0 | 0 | 0 | 1 | 0 | 0 | 1 |
|  |  | 2008 | 2 | 23 | 49 | 28 | 39 | 9 | 25 | 100 | 11 | 19 | 305 |
|  |  | 2009 | 1 | 36 | 40 | 48 | 38 | 15 | 27 | 124 | 3 | 19 | 351 |
|  |  | 2010 | 2 | 34 | 37 | 40 | 48 | 19 | 28 | 130 | 5 | 18 | 361 |
|  |  | 2011 | 1 | 42 | 62 | 41 | 35 | 24 | 30 | 101 | 3 | 24 | 363 |
|  |  | 2012 | 1 | 46 | 69 | 54 | 39 | 16 | 24 | 123 | 13 | 28 | 413 |
|  |  | 2013 | 1 | 42 | 75 | 51 | 49 | 24 | 29 | 105 | 6 | 21 | 403 |
|  |  | 2014 | 2 | 41 | 62 | 69 | 54 | 24 | 29 | 97 | 11 | 19 | 408 |
|  |  | 2015 | 0 | 68 | 69 | 61 | 56 | 29 | 42 | 119 | 10 | 22 | 476 |
|  |  | 2016 | 2 | 34 | 53 | 36 | 43 | 25 | 27 | 87 | 7 | 15 | 329 |
|  |  | 2017 | 0 | 41 | 80 | 71 | 40 | 26 | 24 | 130 | 11 | 11 | 434 |
|  |  | 2018 | 1 | 34 | 84 | 81 | 66 | 19 | 27 | 192 | 13 | 15 | 532 |
|  | Total |  | 13 | 441 | 680 | 580 | 508 | 230 | 312 | 1310 | 93 | 211 | 4378 |
| Total | Year | 1993 | 0 | 0 | 2 | 0 | 0 | 0 | 0 | 0 | 0 | 0 | 2 |
|  |  | 1995 | 0 | 0 | 1 | 0 | 1 | 0 | 0 | 0 | 0 | 0 | 2 |
|  |  | 1996 | 0 | 0 | 1 | 0 | 0 | 0 | 0 | 0 | 0 | 0 | 1 |
|  |  | 1998 | 0 | 1 | 0 | 0 | 0 | 0 | 0 | 1 | 0 | 0 | 2 |
|  |  | 1999 | 0 | 0 | 0 | 0 | 0 | 0 | 0 | 1 | 0 | 0 | 1 |
|  |  | 2000 | 0 | 0 | 0 | 0 | 0 | 0 | 0 | 4 | 0 | 0 | 4 |
|  |  | 2001 | 0 | 0 | 1 | 0 | 1 | 0 | 0 | 1 | 0 | 0 | 3 |
|  |  | 2002 | 0 | 1 | 1 | 0 | 0 | 0 | 0 | 3 | 0 | 0 | 5 |
|  |  | 2003 | 0 | 1 | 3 | 0 | 0 | 0 | 0 | 2 | 1 | 0 | 7 |
|  |  | 2004 | 0 | 0 | 2 | 0 | 0 | 0 | 0 | 2 | 0 | 0 | 4 |
|  |  | 2005 | 0 | 0 | 2 | 0 | 1 | 0 | 0 | 5 | 0 | 0 | 8 |
|  |  | 2006 | 0 | 0 | 0 | 0 | 1 | 1 | 0 | 3 | 0 | 0 | 5 |
|  |  | 2007 | 0 | 0 | 4 | 0 | 0 | 0 | 0 | 3 | 0 | 0 | 7 |
|  |  | 2008 | 10 | 86 | 389 | 152 | 157 | 67 | 78 | 393 | 62 | 65 | 1459 |
|  |  | 2009 | 7 | 86 | 389 | 177 | 141 | 58 | 84 | 379 | 54 | 61 | 1436 |
|  |  | 2010 | 5 | 82 | 333 | 142 | 136 | 66 | 65 | 400 | 49 | 47 | 1325 |
|  |  | 2011 | 9 | 91 | 372 | 160 | 151 | 69 | 65 | 344 | 49 | 56 | 1366 |
|  |  | 2012 | 6 | 101 | 252 | 134 | 119 | 53 | 63 | 351 | 39 | 56 | 1174 |
|  |  | 2013 | 6 | 90 | 238 | 149 | 111 | 47 | 66 | 305 | 26 | 46 | 1084 |
|  |  | 2014 | 5 | 100 | 280 | 172 | 119 | 60 | 63 | 314 | 43 | 40 | 1196 |
|  |  | 2015 | 9 | 131 | 344 | 188 | 157 | 63 | 87 | 380 | 47 | 47 | 1453 |
|  |  | 2016 | 8 | 112 | 319 | 156 | 145 | 72 | 68 | 341 | 52 | 49 | 1322 |
|  |  | 2017 | 6 | 108 | 371 | 195 | 125 | 83 | 85 | 406 | 41 | 39 | 1459 |
|  |  | 2018 | 10 | 100 | 444 | 212 | 177 | 81 | 83 | 484 | 63 | 50 | 1704 |
|  | Total |  | 81 | 1090 | 3748 | 1837 | 1542 | 720 | 807 | 4122 | 526 | 556 | 15029 |
